# Oral Manifestations of COVID-19: Updated Systematic Review With Meta-Analysis

**DOI:** 10.3389/fmed.2021.726753

**Published:** 2021-08-25

**Authors:** Javier Aragoneses, Ana Suárez, Juan Algar, Cinthia Rodríguez, Nansi López-Valverde, Juan Manuel Aragoneses

**Affiliations:** ^1^Department of Medicine and Medical Specialties, Faculty of Medicine and Health Sciences, University of Alcalá, Alcalá de Henares, Spain; ^2^Department of Preclinical Dentistry, School of Biomedical Sciences, Universidad Europea de Madrid, Madrid, Spain; ^3^Department of Clinical Dentistry, School of Biomedical Sciences, Universidad Europea de Madrid, Madrid, Spain; ^4^Department of Dentistry, Federico Henriquez y Carvajal University, Santo Domingo, Dominican Republic; ^5^Department of Surgery, Instituto de Investigación Biomédica de Salamanca (IBSAL), University of Salamanca, Salamanca, Spain; ^6^Dean of the Faculty of Dentistry, Universidad Alfonso X El Sabio, Madrid, Spain

**Keywords:** oral manifestations, oral pathology, oral diseases, COVID-19, SARS-CoV-2, systematic review, meta-analysis

## Abstract

There is increasing evidence for oral lesions and manifestations of COVID-19. The aim of this meta-analysis was to investigate the types of oral manifestations of COVID-19 and their prevalence. PubMed/Medline, Scopus, Web of Science, and Google Scholar databases were used to search for publications on oral manifestations in patients with PCR-confirmed COVID-19. A total of 310 records were selected, and 74 were included. Oral lesions in COVID-19 were classified according to their etiologies, including iatrogenic lesions caused by intubation and opportunistic infections. Of the included studies, 35 reported oral lesions probably caused by severe acute respiratory syndrome-Coronavirus-2 (SARS-CoV-2) infection. Meta-analysis of prevalence data on oral manifestations and aphthous lesions indicated high heterogeneity, while meta-analysis of xerostomia prevalence data revealed a pooled prevalence, with considerable heterogeneity. In conclusion, the meta-analysis yielded high heterogeneity between studies: oral lesions yielded a prevalence of 0.33 (95% CI 0.11–0.60), xerostomia lesions a prevalence of 0.44 (95% CI 0.36–0.52) and aphthous lesions 0.10 (95% CI 0.01–0.24). In addition, a gap in the evidence regarding the prevalence of oral lesions in COVID-19 was identified and the need for further observational studies focusing on this issue and on the causal relationships between oral lesions and COVID-19 was highlighted.

## Introduction

In a span of a few months, coronavirus disease 2019 (COVID-19) has developed into a full-scale global pandemic of epic proportions that affects all age groups with considerable mortality, various sequelae in survivors, and grave socio-economic impact on society (1, 2). Involvement of multiple body systems and organs, including the lung, gastrointestinal tract, liver. blood vessels, heart, nervous system, and kidneys, has been reported in patients with COVID-19 (1). The varied presentations of this disease are under intense study in the hope of better understanding its pathogenesis.

Numerous oral lesions have been reported in patients with COVID-19 (3–8). These manifestations include a wide and varied range, from the inflammation of the papillae of Wharton's duct to plaques on the tongue; however, the exact relationship between many of these lesions and pathologic processes of the severe acute respiratory syndrome-coronavirus-2 (SARS-CoV-2) infection is still a subject of investigation (9). Some of these lesions can clearly be attributed to causes other than the direct effects of SARS-CoV-2 infection. Drug reactions and lesions caused by prolonged intubation are in this group. Moreover, given the sheer number of people afflicted with COVID-19, it is expected that some of the oral lesions seen in these patients are coincidental. Nonetheless, there is growing evidence that a substantial number of oral lesions in COVID-19 are directly related to the pathological processes of the disease (10).

Considering that systematic reviews are an essential tool for synthesizing available scientific information and identifying areas of uncertainty where research is crucial, the present systematic review and meta-analysis (SRMA) aimed to investigate the oral manifestations of COVID-19. A better understanding of the oral manifestations of COVID-19 could help elucidate the pathologic processes involved in this infection and prepare the physician, dentist and health care personnel to cope with the disease. Taste disturbance, which is generally described in conjunction with anosmia, is a prime symptom of COVID-19 (11). This symptom was not addressed in the present review as it clearly surpasses the confines of the oral cavity and involves the sense of smelling.

## Materials and Methods

The present systematic review and meta-analysis (SRMA) follows the guidelines of the Preferred Reporting Items for Systematic Reviews and Meta-analyses (PRISMA) Statement (12).

### Eligibility Criteria

Methodological guidance from the Joanna Briggs Institute on the critical appraisal of prevalence studies and studies of etiology were followed in determining the eligibility criteria (13, 14), and participants, exposure, outcome, study design, as well as context, were taken into consideration. Inclusion criteria encompassed studies reporting oral lesions in patients with PCR-confirmed COVID-19 diagnosis. The primary outcome of interest was the prevalence of oral manifestations of COVID-19. Observational studies, including prevalence studies, case series, and case reports, were included in the review. As for the exclusion criteria, taste disturbances were not included in the review. In studies of mucocutaneous lesions in COVID-19, only cases with oral involvement were included. The eligibility criteria were determined through discussion and consensus among the authors.

### Search Protocol

The eligibility criteria described above were used to design specific search strategies for databases. PubMed/Medline, Scopus, Web of Science, and Google Scholar databases were searched for published and in publication records from December 1, 2019, to March 10, 2021. No language restrictions were applied. The search strategies for these databases are given in the Supplementary Materials 1–4. Moreover, we manually searched the references of review articles on the subject to identify further relevant papers.

### Study Selection

Duplicate records were removed from the collated search results, and titles and abstracts of the remaining records were screened. Records that did not meet the eligibility criteria were discarded. The full texts of the remaining articles were examined, and, again, those that failed to satisfy the eligibility criteria were excluded. Papers were selected in parallel, but independently, by JA, AS and JMA and the reviewers discussed discrepancies in the results and reached a consensus.

### Data Collection

Relevant information on geographical location and design of the study and data on the prevalence of oral lesions and their subgroups were extracted from prevalence studies. In case reports and case series, information on geographical location and design of the study, participants' sex and age and general and oral signs and symptoms were extracted. Specific forms designed by the reviewers were used in data extraction.

### Risk of Bias Assessment

The Joanna Briggs Institute's Critical Appraisal Checklists for Studies Reporting Prevalence Data was used to assess the risk of bias in prevalence studies. For assessing case reports and case series on lesions caused by SARS-CoV2 infection, the Joanna Briggs Institute's Critical Appraisal Checklist for Case Reports and Case series were used, respectively (15). The risk of bias in a study was considered high if the “yes” score was 49% or lower. Studies with a score between 50 and 69% were considered at moderate risk and those with a score of 70% or higher at low risk of bias. The reviewers could override the evaluation and modify the risk of bias by consensus, providing specific reasons.

### Data Synthesis

The main outcome of interest was the prevalence of oral manifestations of COVID-19. Available data on the prevalence of oral lesions in general and their subgroups were synthesized, and a meta-analysis of prevalence was performed on them. Oral lesions in patients with COVID-19, reported in case reports and case series, were classified according to their putative etiologies, e.g.,: oral manifestations caused by SARS-CoV-2 infection, opportunistic co-infections, and iatrogenic lesions. For oral manifestations caused by SARS-CoV-2 infection, relevant data on patient and disease characteristics and oral manifestations were summarized in table form and qualitative synthesis was performed. Heterogeneity among the prevalence studies was computed using the Q statistic and the *I*^2^ index. The random effects model was used in the meta-analysis. The meta-analysis of prevalence was performed using the approach proposed by Barendregt et al. (16). Double arcsine transformation was employed to stabilize the variance, and overall prevalence (95% CI) was calculated. The leave-one-out sensitivity analysis was performed to explore whether the inclusion of the studies at high risk of bias in the meta-analysis distorted the results. Meta-analysis was performed using MetaLX (Version 5.3) software for meta-analysis in Microsoft Excel. The significance level was set at *P* < 0.05. Meta-regression analysis of the prevalence studies was performed with three moderator variables of the geographical location of the study, study design, and risk of bias. The MARegresData function in MetaXL was used to create regression datasets for studies on the prevalence of oral lesions in total, aphthous lesions, and xerostomia. The regression data set contained transformed effect sizes with standard error and variance, un-transformed effect sizes with lower and upper CIs, and normalized weight of studies. These data were transferred to STATA statistical software, version 16 and analyzed using the “Regress” command and robust (Huber-Eicker-White-sandwich) standard error estimation. *P*-values < 0.05 were considered significant.

### The Certainty of Evidence Assessment

The certainty of evidence in prevalence studies was assessed using the Grading of Recommendations, Assessment, Development and Evaluations (GRADE) approach (17). The evidence was rated according to the level of concern in the domains of the risk of bias, inconsistency, imprecision, indirectness, and publication bias. Publication bias was assessed by drawing funnel plots and visually inspecting them.

## Results

### Characteristics of the Studies

**Figure 1:**
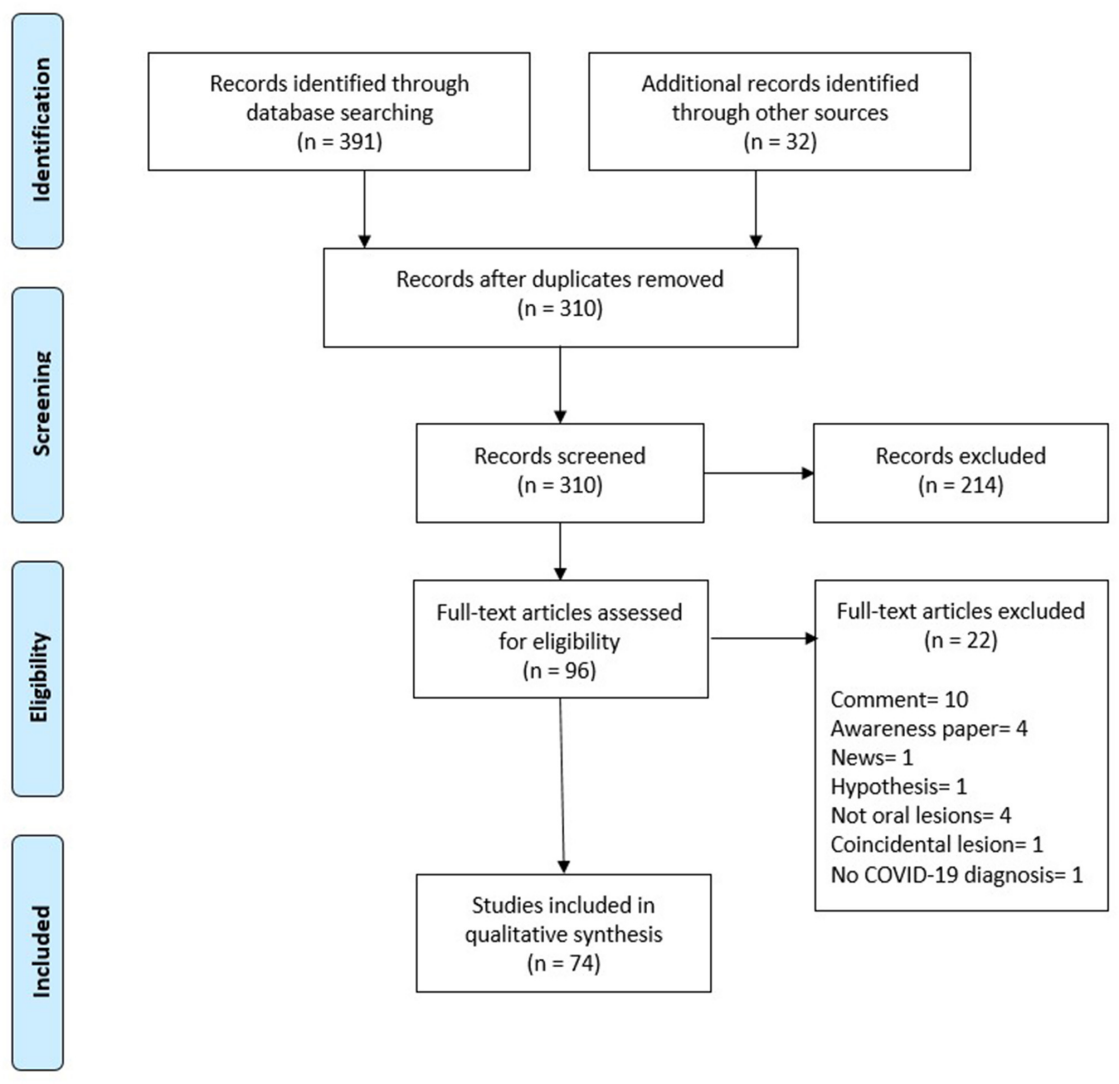
Flowchart.

As of March 10, 2021, 391 records were identified. An additional 32 records were identified by searching references from relevant review articles. After removing duplicate records, 310 entries remained. The titles and abstracts of these records were studied, and 214 records were excluded at this stage. A total of 96 articles were searched for eligibility, excluding 22 studies at this stage because they either did not include reports of oral manifestations in patients with COVID-19 or reported coincidental oral lesions that were clearly not caused by COVID-19. One excluded article reported oral lesions in a patient with suspected COVID-19 without PCR confirmation of disease. Thus, 74 studies on oral manifestations in patients with COVID-19 were selected for review. Of these 74 records, 10 were prevalence studies and the remaining 64 were case reports and case series on oral manifestations (Figure 1, Flowchart).

A total of 10 studies with information on the prevalence of oral manifestations in COVID-19 were identified (18–27), with a total of patients included in these studies was 3,519. Three reported the number of patients with oral lesions in general (18, 22, 25), four gave the number of patients with xerostomia (18, 20, 21, 23), and four mentioned the number of patients with aphthous lesions (19, 22, 25, 27). A total of 64 records retrieved were case reports and case series of oral manifestations in COVID-19. These cases were classified according to their presumed etiologies into three main categories and eight etiologic groups. In the category of oral manifestations with probable causes other than SARS-CoV-2 infection, iatrogenic lesions (lesions from intubation and other invasive procedures) (28, 29), drug reactions (30), and opportunistic co-infections with Candida albicans (31–34) or Herpes simplex (35–39) were included. The second category comprised multiorgan syndromes with variable oral manifestation. Clinical entities in this group usually involve several organ systems, but the oral manifestation in them is not constant and is observed in only a percentage of patients. Multisystem inflammatory syndrome and Kawasaki-like disease (40–53), as well as oral lesions that are part of other multiorgan involvement (urticaria and angioedema with mouth involvement) (54). he third group included oral manifestations caused directly by SARS-CoV-2 infection, such as relevant oral lesions (7, 55–85) and salivary gland involvement (86–89); 35 studies reported cases and case series with oral lesions in COVID-19 with no apparent non-COVID-19 causes. The characteristics of these studies are summarized in Tables 1–3.

**Table 1:** Characteristics of prevalence studies.

**References (location)**	**Study type & description**	**Sample size**	**Oral lesions in total**	**Aphthous lesions**	**Xerostomia**	**Risk of bias**
Abubakr et al. (18) (Egypt)	Questionnaire; oral complaints	573	117	N/A	273	Moderate
Askin et al. (19) (Turkey)	Prospective observational; cutaneous manifestations	210	N/A	3	N/A	High
Biadsee et al. (20) (Israel)	Questionnaire; olfactory and oral manifestation	140	N/A	N/A	72	Moderate
Fantozzi et al. (21) (Italy)	Questionnaire; xerostomia	111	N/A	N/A	51	Moderate
Fidan et al. (22) (Turkey)	Prospective observational; oral lesions	74	58	27	N/A	Low
Gherlone et al. (23) (Italy)	Retrospective and prospective cohort study; salivary gland ectasia and oral disease	122	N/A	N/A	37	Low
Khabadze et al. (24) (Russia)	Retrospective study; Changes in oral mucosa	90	N/A	N/A	N/A	High
Nuno-Gonzalez et al. (25) (Spain)	Cross-sectional study; mucocutaneous manifestations and oral and palmoplantar findings	666	78	46	N/A	Moderate
Rekhtman et al. (26) (USA)	Prospective cohort study; eruptions	296	N/A	N/A	N/A	High
Riad et al. (27) (Czech Republic)	Retrospective	1,237	N/A	21	N/A	Moderate

**Table 2:** Classification of oral manifestations (except taste alterations) in patients with COVID-19 according to etiology.

**Groups of oral manifestations**	**Examples**
**Oral manifestations with likely direct causes other than SARS-CoV-2 infection**	
Iatrogenic lesions	Intubation injuries
Drug reactions	
Opportunistic coinfections	Candidiasis, Herpes simplex virus infections
**Multi-organ involvement with variable oral manifestation**	
Oral lesions in multisystem inflammatory syndrome and Kawasaki-like disease	
Oral lesion as part of other multi-organ involvements	Urticaria and angioedema involving the mouth
**Oral manifestations probably caused directly by SARS-CoV-2 infection**	
Lesions with SARS-CoV-2 infection as the likely direct cause	Aphthous lesions, oral ulcers
Salivary gland involvement	Sialadenitis
Xerostomia	

**Table 3:** Characteristics of oral manifestations reported in case reports and case series.

**References (location)**	**Cases**	**Sex/age (years)**	**Oral manifestations**	**General findings**
Afsal et al. (55) (India)	3 cases	NA	Inflammation of the papillae of Wharton's Duct; hyposalivation	Mild fever, myalgia, throat pain
Aghazadeh et al. (56) (Iran)	1 case	F/9	Herpetiform eruption comprising vesicles and erosions on the lips, anterior tongue, and buccal mucosa	High fever, severe weakness, loss of appetite, abdominal pain, diarrhea; dry cough, shortness of breath, tachypnea, hypoxia; deep red, edematous papules and plaques on the dorsal aspect of hands and feet
Al-Khanati et al. (57) (Syria)	1 case	M/24	Two aphthous-like ulcers on the lower lip with pain; areas of overgrowth and absence of filiform papillae in the tongue with burning sensation; inflammatory and purulent areas on the pharyngeal wall	Very severe pain all over the body, especially in the shoulders, lower back, and knees; very severe headache, dizziness, loss of appetite, nausea, high temperature
Amorim Dos Santos et al. (58) (Brazil)	1 case	M/67	Persistent white plaque and multiple pinpoint yellowish ulcers- similar to herpetic recurrent oral lesions- on the dorsal part of the tongue; a 1 cm nodule on the lower lip suggestive of fibroma; extremely viscous saliva; plaque resolved with treatment and was replaced by an asymptomatic geographical tongue	Respiratory symptoms, progressive dyspnea on exertion, fever, diarrhea, hypogeusia

### Risk of Bias Assessment

The risk of bias was assessed using Joanna Briggs Institute's Critical Appraisal Checklists for Studies Reporting Prevalence Data, and then, the reviewers examined and finalized the results of the assessment. Overall, 10 prevalence studies were assessed (18–27). In two study, the risk of bias was determined as low (22, 23), in five studies as moderate (18, 20, 25, 27), and in three as high (19, 24, 26). The risk of bias assessment and the reasons for the upgraded assessments are given in the Supplementary Material 5.

### Results of Meta-Analysis

Table 1 shows 10 studies addressing the prevalence of oral lesions in COVID-19. Data on the prevalence of oral lesions in general, xerostomia, and aphthous lesions were meta-analyzed. For each of these three variables, studies reporting data on that variable were included in the relevant calculations. In groups that contained studies at high risk of bias, the meta-analysis was repeated after removing those studies. Of 10 studies, three reported data on more than one variable (18, 22, 25) and were accordingly included in more than one analysis; while two studies did not report data for any of the three variables (24, 26) and therefore, were not included in the calculations.

**Figure 2:**
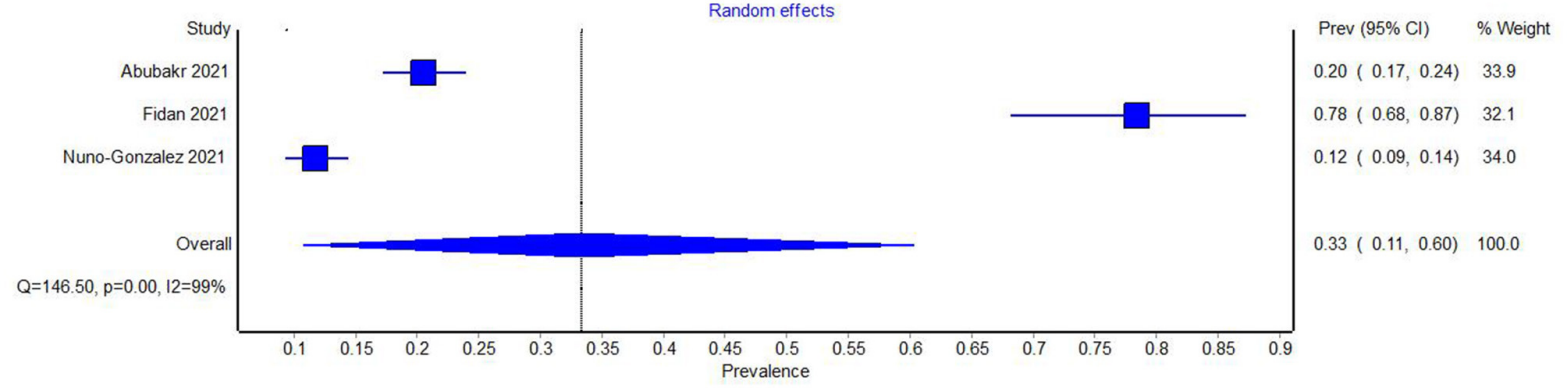
Forest plot showing the pooled prevalence of oral lesions in general in COVID-19.

Among prevalence studies, three presented data on the number of patients with oral lesions in general (18, 22, 25). The heterogeneity of the studies was examined using Cochran's Q test and the *I*^2^ index. Both these measures indicated very large heterogeneity among the studies (*Q* = 146.50, *p* = 0.00, *I*^2^ = 99%). The Meta-analysis of the prevalence of oral lesions in general in COVID-19 yielded a prevalence of 0.33 (95% CI 0.11–0.60) (Figure 2). The sensitivity study did not lead to a significant difference in the results.

**Figure 3:**
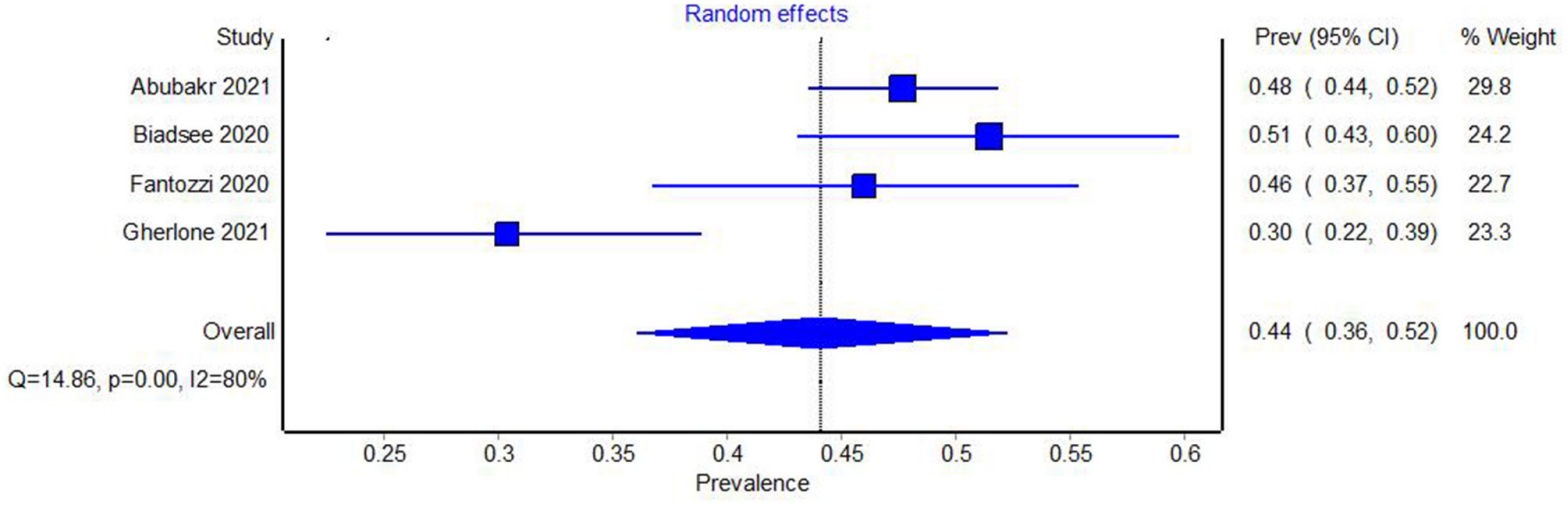
Forest plot showing the pooled prevalence of xerostomia in COVID-19.

A total of four studies reported the number of patients with xerostomia in the sample under study (18, 20, 21, 23). Cochran's Q test and the *I*^2^ index were used to examine the heterogeneity of the studies, both of which revealed large heterogeneity among the studies (Q = 14.86, *p* = 0.00, *I*^2^ = 80%). The Meta-analysis of the prevalence of xerostomia in COVID-19 yielded a prevalence of 0.44 (95% CI 0.36–0.52) (Figure 3).

**Figure 4:**
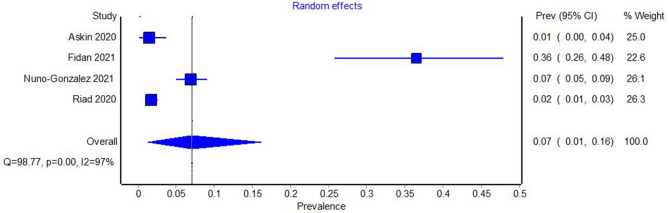
Forest plot showing the pooled prevalence of aphthous oral lesions in COVID-19, with studies at high risk of bias included.

Among prevalence studies, three reported the number of patients with aphthous lesions (19, 22, 25), one of which had been evaluated as being at high risk of bias (19). The heterogeneity of the studies, including the one at the high risk of bias, was examined using Cochran's Q test and the *I*^2^ index. Both these measures indicated very large heterogeneity among the studies (Q = 98.77, *p* = 0.00, *I*^2^ = 97%). The Meta-analysis yielded a prevalence of 0.07 (95% CI 0.01–0.16) (Figure 4).

**Figure 5:**
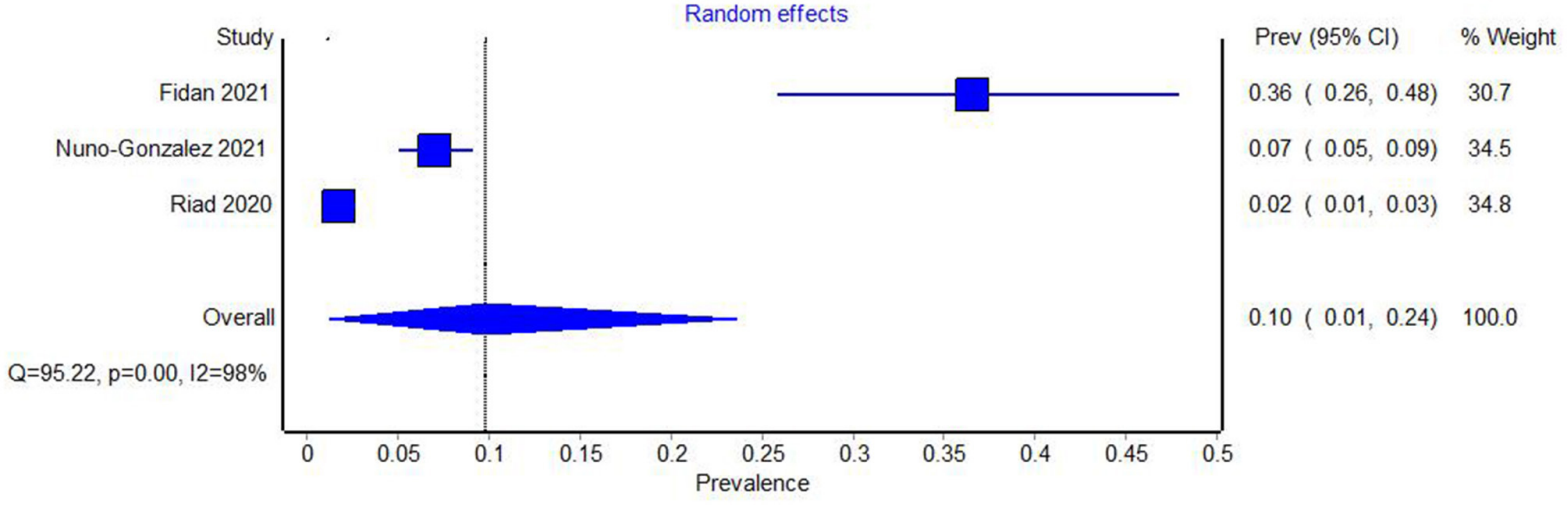
Forest plot showing the pooled prevalence of aphthous oral lesions in COVID-19 with studies at high risk of bias excluded.

The study with a high risk of bias was eliminated from the analysis and the heterogeneity tests and meta-analysis were repeated. Again, high heterogeneity between studies was evident (Q = 95.22, *p* = 0.00. *I*^2^ = 98%). The prevalence of aphthous lesions in COVID-19 was 0.10 (95% CI = 0.01–0.24) (Figure 5).

In the meta-regression analysis of prevalence studies, three putative moderating variables were studied: geographical location of the study, study design, and risk of bias. No significant relationship was detected between these variables and the prevalence estimate for oral lesions in total or the prevalence estimate for aphthous lesions. In xerostomia studies, variables of study design and risk of bias showed an effect on the prevalence estimate (R-squared = 0.9417, *p* < 0.003).

**Figure 6:**
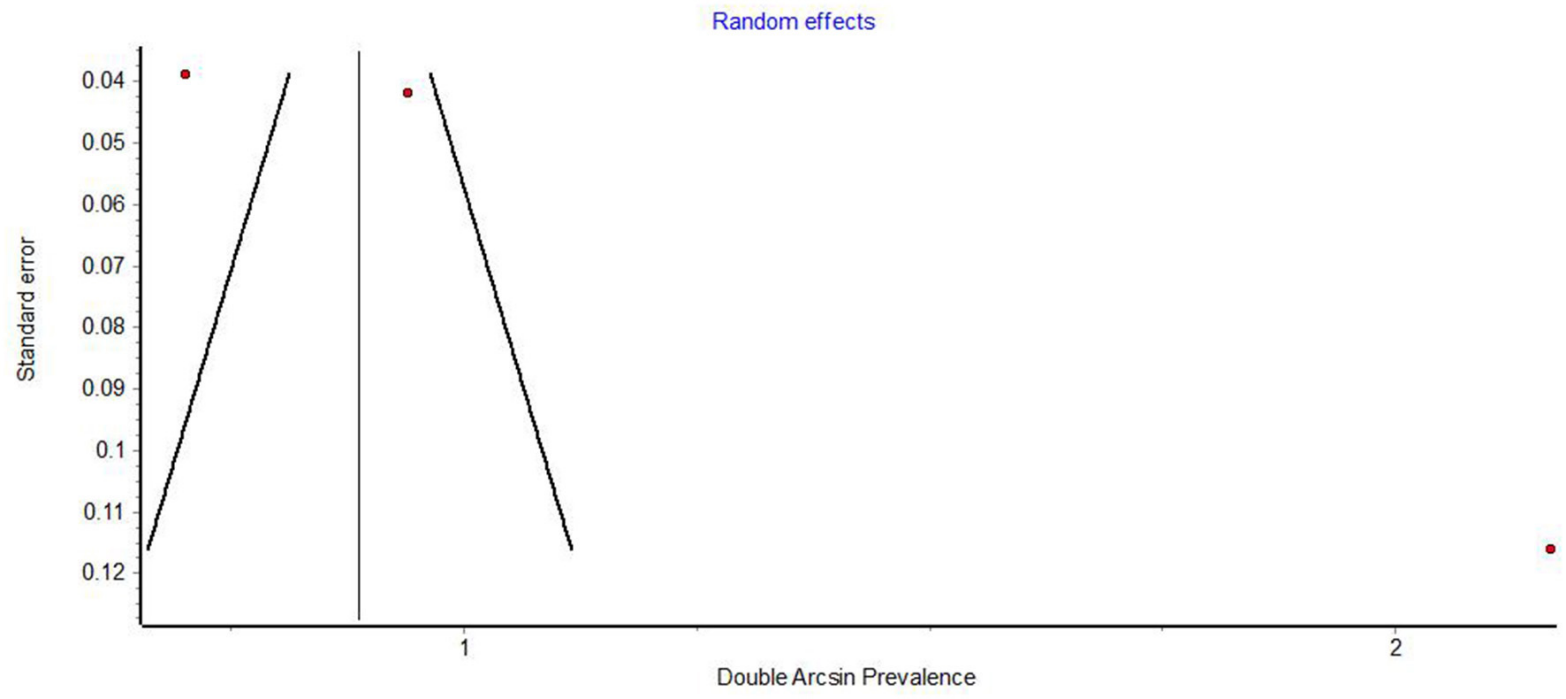
Funnel plot of studies on the prevalence of oral lesions in general.

**Figure 7:**
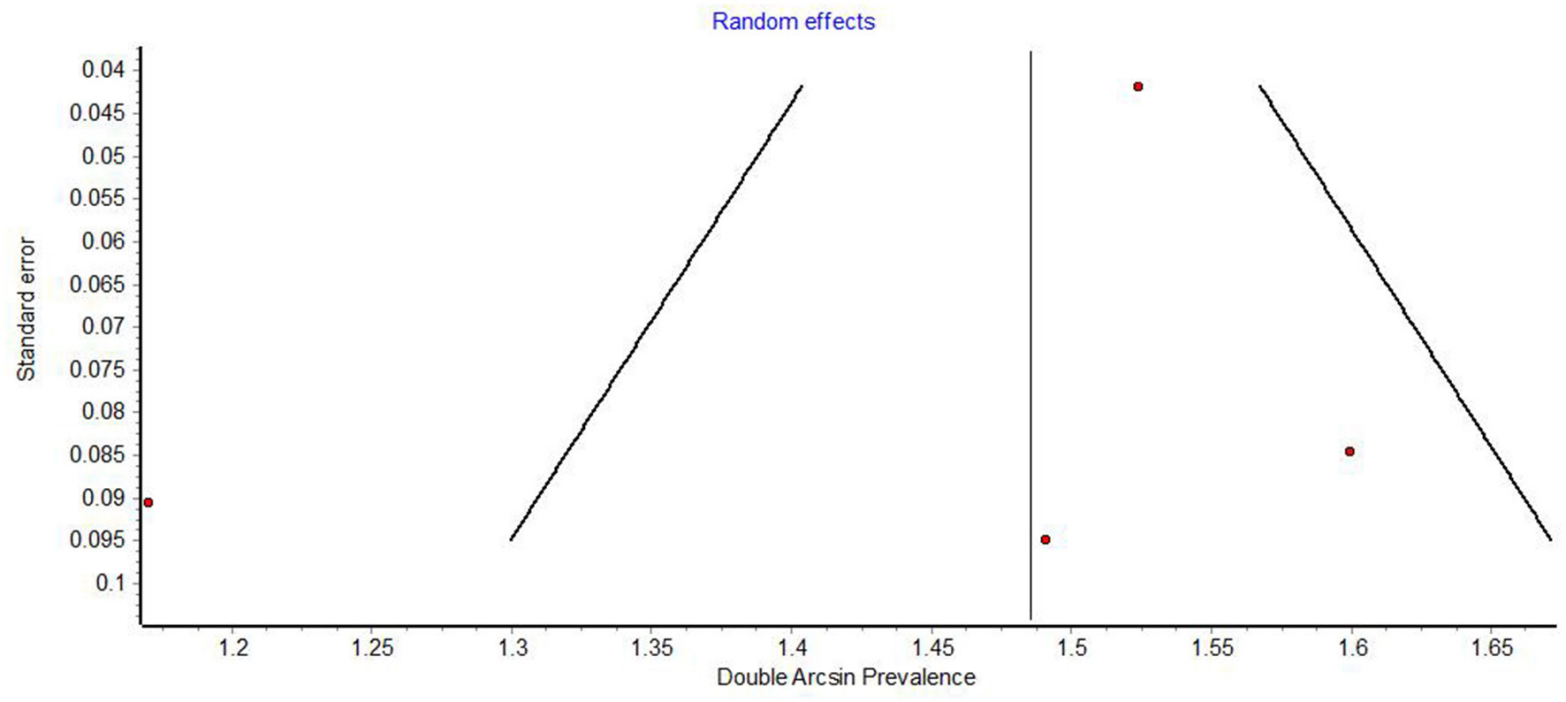
Funnel plot of studies on the prevalence of xerostomia.

**Figure 8:**
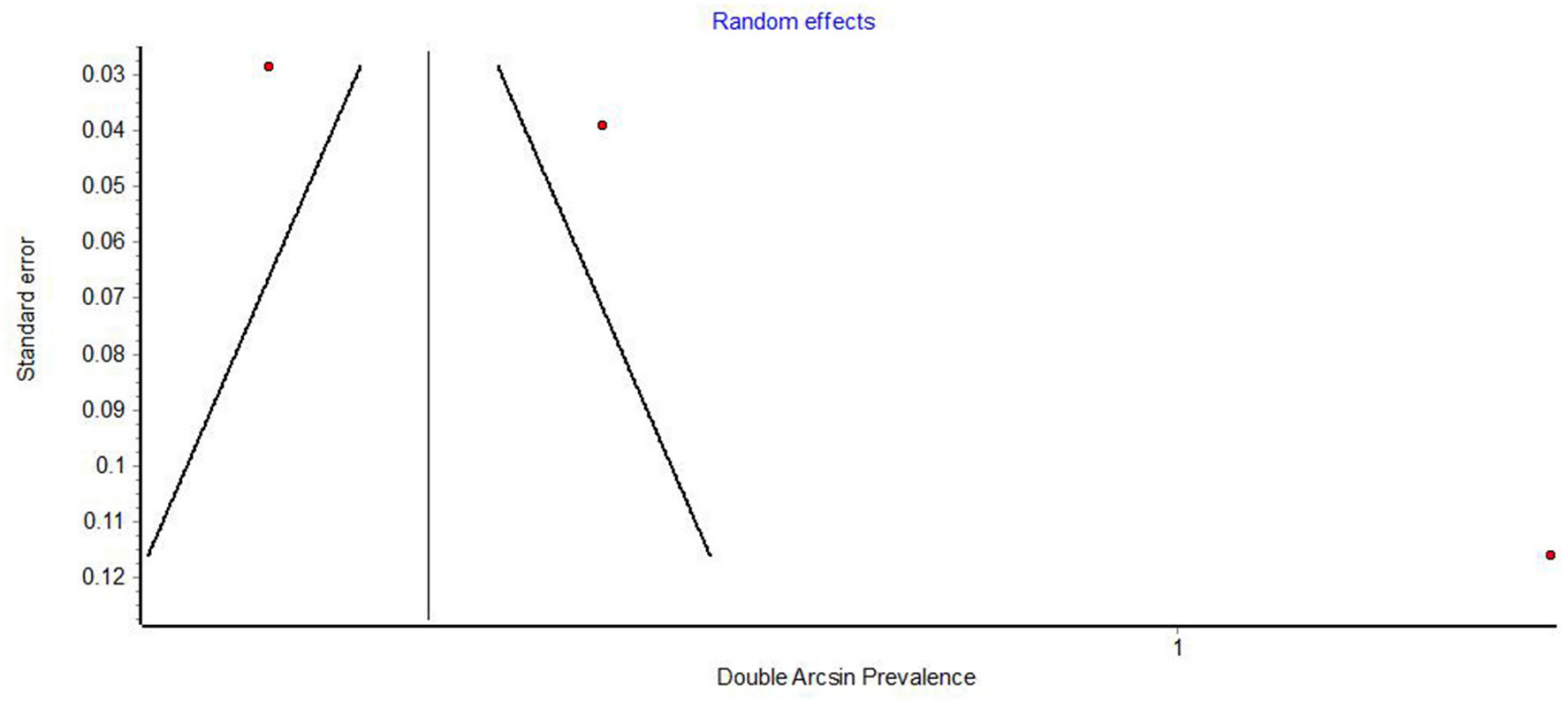
Funnel plot of studies on the prevalence of aphthous lesions.

Funnel plots demonstrated obvious asymmetry, indicating the presence of high publication bias (Figures 6–8).

The GRADE tool assessed the prevalence of oral lesions in general and aphthous lesions as low, while the prevalence of xerostomia was assessed as moderate (Table 4).

**Table 4:** Grades of evidence (GRADE tool).

**Outcome**	**Result**	**Number of participants (studies)**	**Certainty of the evidence (GRADE)**	**References**
Oral lesions in general	0.33 (95% CI 0.11–0.60)	1,313 (3)	**⊕⊕⊖⊖** Low	(18, 22, 25)
Xerostomia	0.48 (95% CI 0.45–0.51)	946 (4)	**⊕⊕⊕⊖** Moderate	(18, 19, 21, 23)
Aphthous lesions	0.10 (95% CI= 0.01–0.24)	2,187 (4)	**⊕⊕⊖⊖** Low	(19, 22, 25, 27)

## Discussion

To date, very few studies have been published that directly address the question of the prevalence of oral manifestations in patients with COVID-19 using adequate detection methods. Much of the data available on the subject is still evidence from studies on mucocutaneous involvement or data gathered through patient questionnaires. Nonetheless, the SRMA of prevalence data on oral manifestations in COVID-19 at this stage, can identify and define important gaps in evidence and set the stage for further research, and even provide preliminary results of great interest.

The number of studies reporting on the prevalence of oral manifestations in COVID-19 remains very low and the problems mentioned above, make the existing reports at considerable risk of bias. A comprehensive search of the relevant literature identified only 10 primary studies with data on the topic (18–27), with the risk of bias being high in three studies, moderate in four and low in two. Three studies relied on patient-directed questionnaires as the data collection vehicle (18, 20, 21), a fact that is bound to impact the accuracy of the data. In addition, many of the studies did not focus on oral lesions but mentioned these lesions within the framework of a broader study on mucocutaneous lesions. Similarly, some of the prevalence studies focused on a specific type of lesions, e.g., xerostomia or rashes (21, 26), despite the reviewers improved the risk of bias assessed for some of the included studies (19, 23, 24, 26).

On the other hand, the included studies report on the prevalence of oral manifestations of COVID-19 in three categories: oral manifestations in general, xerostomia, and aphthous lesions. In the studies concerning the prevalence of oral lesions in general and aphthous lesions, tests showed a very high degree of heterogeneity- *I*^2^ = 99% for oral lesions in general and *I*^2^ = 98% for aphthous lesions. The meta-analysis of prevalence yielded a prevalence of 0.33 (95% CI 0.11–0.60) for oral lesions in general and 0.10 (95% CI = 0.01–0.24) for aphthous lesions.

These high degrees of heterogeneity reflect the many methodological issues affecting the included studies regarding the prevalence of COVID-19 oral lesions; specific studies on the topic, with sufficient sample sizes and careful examination and classification of oral lesions, are still lacking. Any review of the topic has to rely on obtaining data from studies that either do not focus specifically on oral lesions or collect the information through patient questionnaires. Similarly, the 95% confidence intervals (disproportionately wide) of the two meta-analyses give an idea of the imprecision of the results. Therefore, the results of the meta-analysis of these variables should be viewed with caution and only as preliminary indicators to lay the groundwork for future research. In line with these observations, the GRADE assessment found that the certainty of the evidence for these two indices is low, which means that future research is very likely to change the current estimates.

As for the prevalence of xerostomia, the heterogeneity of the studies was high (*I*^2^ = 80%). In the meta-analysis, the prevalence estimate was 0.44 (95% CI 0.36–0.52). The relatively narrow range of the 95% confidence intervals bolsters the findings. In agreement with these considerations, the GRADE evaluation suggested moderate certainty of evidence in this area.

Meta-regression analysis of prevalence studies was performed in an attempt to explore the possible sources of heterogeneity in the results of these studies. The geographical location of the study, study design, and risk of bias did not show a significant effect on the prevalence estimate for oral lesions in total or the prevalence estimate for aphthous lesions. As for xerostomia, variables of study design and risk of bias showed significant effects on the prevalence estimate (R-squared = 0.9417, *p* < 0.003), emphasizing the need for studies with more robust quality and study design (e.g.,: prospective studies) in the future. In general, meta-regression analysis could not amply explain the sources of heterogeneity in the prevalence studies, presumably because of the paucity of eligible studies in this area.

Funnel plots of prevalence studies demonstrate noticeable asymmetry (Figures 6–8). The asymmetry is in the scatter of small studies and is evident in all three funnel plots, but this scatter is not always right-sided, as would be expected in publication bias. Funnel plots of studies on the prevalence of oral lesions in general (Figure 6) and studies on the prevalence of aphthous lesions (Figure 8) show a right-sided asymmetry, suggesting publication bias; while the funnel plot of studies on the prevalence of xerostomia shows a pronounced asymmetry to the left. This asymmetry may have arisen from inadequate detection methods in the smaller studies. Whatever the reason for this discrepancy in the results regarding xerostomia, the underreporting of this condition in smaller studies impacts the prevalence estimate of xerostomia in COVID-19.

In summary, the SRMA of prevalence data suggests a high prevalence of xerostomia. The mechanisms of this phenomenon in COVID-19 and its possible relationship with the pathologic processes of the disease are currently under discussion (90). In a broader perspective, the relatively high frequency of xerostomia and aphthous lesions in case reports and case series of oral manifestations corroborates the assertion that these two findings occur frequently in patients with COVID-19.

In this SRMA, we have proposed a framework for classifying oral lesions seen in COVID-19 according to their putative etiologies.

Oral manifestations in COVID-19 patients are varied and of diverse etiologies. To embark upon a systematic review of the oral manifestations of COVID-19, the myriad afflictions of the mouth reported in this group of patients should be scrutinized and their possible relationship with this infection examined. In this SRMA, we have proposed a framework for classifying oral lesions seen in COVID-19 according to their putative etiologies.

Many of the oral afflictions seen in COVID-19 patients are not directly caused by SARS-CoV-2 infection and, thus, should not be classified as oral manifestations of this disease. One notable group in this category is iatrogenic complications that occur in the course of the treatment of COVID-19. This group includes lesions caused by mechanical trauma of prolonged intubation and other invasive procedures employed (28, 29). Also, drug reactions presenting in the form of oral lesions have been reported (30), which can be classified in this category.

Another group of oral lesions seen in patients with COVID-19, but not directly related to the pathologic processes of SARS-CoV-2 infection, are opportunistic co-infections that involve the oral cavity. Oral co-infections reported thus far in patients with COVID-19 include candidiasis and Herpes simplex infections (31–39). As in many other debilitating diseases, the occurrence of such opportunistic co-infections is to be expected in COVID-19.

Taste disturbance/anosmia was not addressed in the present review as it clearly surpasses the confines of the oral cavity and involves the sense of smelling. Moreover, some patients with COVID-19 present with distinctive constellations of signs and symptoms that involve several organ systems including the mouth. Notable examples of such constellations are the multisystem inflammatory syndrome and Kawasaki-like disease frequently encountered in children with COVID-19 (39–52). Toxic epidermal necrolysis (TEN) and urticaria and angioedema with mucocutaneous involvement are other examples (54, 91). The presence of oral lesions in these clinical syndromes is variable, and a considerable proportion of the patients afflicted with these syndromes do not have oral lesions. Furthermore, the clinical picture of these syndromes indicates that the underlying pathological processes are much broader than the confines of the oral cavity. Therefore, while the mouth lesions in these syndromes should be noted and described, such oral involvements are better addressed in the context of the relevant clinical syndromes rather than the oral manifestations of COVID-19.

After the above-mentioned categories are excluded, a considerable body of evidence on the oral manifestations of COVID-19 remains (7, 55–89). This category includes oral lesions and salivary gland involvement. It is highly probable that many of these manifestations are directly caused by SARS-CoV-2 infection. For all these reasons, case reports and case series related to this category are discussed in more detail here. The 35 Case report and case series papers are from various locations spanning the continents of Africa, Asia, Europe, and Americas. Brazil has the highest number of publications (seven papers). A total of 236 have been described in these papers. Of the patients, 119 are female and 113 are male. In the remaining four patients, gender has not been specified. The patients range from 9 to 88 years old and represent every age group in between. The oral lesions in the patients are highly varied, with the most reported being ulcerations in various parts of the oral cavity in 12 papers. The tongue is the most frequently afflicted part of the oral cavity, reported in 20 papers.

Oral lesions likely caused by SARS-CoV2 infection present a very heterogeneous group affecting almost the entire oral cavity. The patterns of occurrence of these manifestations are gradually emerging through case reports and other observational studies, and their probable causal relationship to SARS-CoV2 remains under study. Several groups of viruses, such as herpesviruses and human papillomaviruses, are known to cause oral lesions (11, 92), and the potential of SARS-CoV2 coronavirus to cause oral lesions should be thoroughly investigated. However, the role of coincidence, comorbidities and opportunistic co-infections in the occurrence of some of these lesions cannot be ruled out at this time.

To date, no consensus has been reached on the causal relationship between SARS-CoV2 infection and oral manifestations of COVID-19 and very few studies have focused specifically on the question of the prevalence of oral lesions in COVID-19, therefore, any decision to include a particular group of lesions in this category is, to some extent, subjective.

Numerous reviews have addressed the question of oral manifestations in patients with COVID-19 (3–8) but, in general, these studies have reviewed case reports and case series and discussed the association of SARS-CoV2 infection with these manifestations. In this sense, the present RSMA agrees with previous reviews and studies, presenting, in addition, a meta-analysis on the prevalence of oral lesions in COVID-19, which will pave the way for further studies on this pathology.

The present paper reflects certain limitations. COVID-19 is a novel and rapidly evolving disease and many of its features, including the oral manifestations associated with it, are still under investigation. As yet, no consensus has been reached regarding the nature and classification of the oral lesions in COVID-19 and their causative relationship with the diseases process. This limitation is reflected in the systematic reviews of the subject such as the present paper. Moreover, there is a marked paucity of high-quality studies on the prevalence of these oral manifestations, and this limitation affects the prevalence estimates presented in this meta-analysis. The above limitations are reflected in the heterogeneity in the results of the included studies.

## Conclusions

Case reports and case series describe a relatively high frequency of xerostomia and aphthous lesions in patients with COVID-19. There is a markedly paucity of specific studies on the subject, with sufficient sample sizes and careful examination and classification of oral lesions; therefore, it is suggested that more studies of robust, prospective design are conducted on oral lesions of COVID-19, with particular attention to the classification of these lesions.

## Data Availability Statement

The original contributions presented in the study are included in the article/Supplementary Material, further inquiries can be directed to the corresponding author/s.

## Author Contributions

JAr, AS, and JMA: conceptualization, formal analysis, writing original draft, and supervision. JAr: methodology. NL-V: software. JAr, AS, JMA, JAl, CR, and NL-V: validation and review and editing. All authors contributed to the article and approved the submitted version.

## Conflict of Interest

The authors declare that the research was conducted in the absence of any commercial or financial relationships that could be construed as a potential conflict of interest.

## Publisher's Note

All claims expressed in this article are solely those of the authors and do not necessarily represent those of their affiliated organizations, or those of the publisher, the editors and the reviewers. Any product that may be evaluated in this article, or claim that may be made by its manufacturer, is not guaranteed or endorsed by the publisher.
