# Supplementary material for: Oral Manifestations of COVID-19: Updated Systematic Review With Meta-Analysis
Source: Front Med (Lausanne). 2021 Aug 25;8:726753. doi: 10.3389/fmed.2021.726753 (PMC8424005; doi:10.3389/fmed.2021.726753)
Supplement: Supplementary file 1 [file Data_Sheet_1.pdf]

Supplementary Material

S1- PubMed search strategy

(2019nCov*[ti] OR 2019-nCoV*[ti] OR SARS-Cov*[ti] OR covid*[ti] OR Wuhan*[ti] OR SARS-CoV-2[mh] OR COVID-19[mh]) AND (“oral manifestation*”[tiab] OR “oral patholog*”[tiab] OR “mouth disease*”[tiab] OR "oral disease*"[tiab] OR “oral le-sion*”[tiab] OR "oral complication*”[tiab] OR “oral change*”[tiab] OR "oral mucosal disease*"[tiab] OR "oral mucosal lesion*"[tiab] OR "oral mucosal complication*”[tiab] OR “oral mucosal change*”[tiab] OR "mucocutaneous disease*"[tiab] OR "mucocutaneous lesion*"[tiab] OR "mucocutaneous complication*”[tiab] OR “mucocutaneous change*”[tiab] OR Oral Manifestations[mh] OR Pathology, Oral[mh] OR Mouth Diseas-es[mh])
