# Supplementary material for: Oral Manifestations of COVID-19: Updated Systematic Review With Meta-Analysis
Source: Front Med (Lausanne). 2021 Aug 25;8:726753. doi: 10.3389/fmed.2021.726753 (PMC8424005; doi:10.3389/fmed.2021.726753)
Supplement: Supplementary file 2 [file Data_Sheet_2.pdf]

Supplementary Material

S2- Scopus search strategy

(TITLE(Wuhan) OR TITLE(2019nCov*) OR TITLE(2019-nCoV*) OR TITLE(SARS-Cov*) OR TITLE(covid*)) AND (TI-TLE-ABS-KEY(“oral manifestation*”) OR TITLE-ABS-KEY(“oral patholog*”) OR TI-TLE-ABS-KEY(“mouth disease*”) OR TITLE-ABS-KEY("oral disease*") OR TI-TLE-ABS-KEY(“oral lesion*”) OR TITLE-ABS-KEY("oral complication*”) OR TI-TLE-ABS-KEY(“oral change*”) OR TITLE-ABS-KEY("oral mucosal disease*") OR TI-TLE-ABS-KEY("oral mucosal lesion*") OR TITLE-ABS-KEY("oral mucosal complica-tion*”) OR TITLE-ABS-KEY(“oral mucosal change*”) OR TI-TLE-ABS-KEY("mucocutaneous disease*") OR TITLE-ABS-KEY("mucocutaneous le-sion*") OR TITLE-ABS-KEY("mucocutaneous complication*”) OR TITLE-ABS-KEY(“mucocutaneous change*”))
