# Supplementary material for: Oral Manifestations of COVID-19: Updated Systematic Review With Meta-Analysis
Source: Front Med (Lausanne). 2021 Aug 25;8:726753. doi: 10.3389/fmed.2021.726753 (PMC8424005; doi:10.3389/fmed.2021.726753)
Supplement: Supplementary file 3 [file Data_Sheet_3.pdf]

Supplementary Material

S3- Web of Science search strategy

TI=(Wuhan* OR 2019nCov* OR 2019-nCoV* OR SARS-Cov* OR covid* OR SARS-CoV-2) AND AB=("oral manifestation*" OR "oral patholog*" OR "mouth disease*" OR "oral disease*" OR "oral lesion*" OR "oral complication*" OR "oral change*" OR "oral mucosal disease*" OR "oral mucosal lesion*" OR "oral mucosal complication*" OR "oral mucosal change*" OR "mucocutaneous disease*" OR "mucocutaneous lesion*" OR "mucocutaneous complication*" OR "mucocutaneous change*")
