# Supplementary material for: Oral Manifestations of COVID-19: Updated Systematic Review With Meta-Analysis
Source: Front Med (Lausanne). 2021 Aug 25;8:726753. doi: 10.3389/fmed.2021.726753 (PMC8424005; doi:10.3389/fmed.2021.726753)
Supplement: Supplementary file 4 [file Data_Sheet_4.pdf]

Supplementary Material

S4- Google Scholar search strategy

allintitle: COVID 19 AND oral manifestations
