# Supplementary material for: Oral Manifestations of COVID-19: Updated Systematic Review With Meta-Analysis
Source: Front Med (Lausanne). 2021 Aug 25;8:726753. doi: 10.3389/fmed.2021.726753 (PMC8424005; doi:10.3389/fmed.2021.726753)
Supplement: Supplementary file 5 [file Data_Sheet_5.pdf]

Supplementary Material

S5- Critical appraisal of studies reporting prevalence data

Abubakr 2021

	Yes	No	Unclear	Not applicable
1. Was the sample frame appropriate to address the target population?		X		
2. Were study participants sampled in an appropriate way?	X			
3. Was the sample size adequate?	X			
4. Were the study subjects and the setting described in detail?	X			
5. Was the data analysis conducted with sufficient coverage of the identified sample?	X			
6. Were valid methods used for the identification of the condition?		X		
7. Was the condition measured in a standard, reliable way for all participants?		X		
8. Was there appropriate statistical analysis?				X
9. Was the response rate adequate, and if not, was the low response rate managed appropriately?	X			

Overall appraisal: Include X Exclude Seek further info

Comments (Including reason for exclusion):

Askin 2020

	Yes	No	Unclear	Not applicable
1. Was the sample frame appropriate to address the target population?	X			
2. Were study participants sampled in an appropriate way?	X			
3. Was the sample size adequate?				
4. Were the study subjects and the setting described in detail?	X			
5. Was the data analysis conducted with sufficient coverage of the identified sample?	X			
6. Were valid methods used for the identification of the condition?		X		
7. Was the condition measured in a standard, reliable way for all participants?		X		
8. Was there appropriate statistical analysis?				X

9. Was the response rate adequate, and if not, was the low response rate managed appropriately?	X			

Overall appraisal: Include Exclude X Seek further info

Comments (Including reason for exclusion): The study aimed to investigate cutaneous manifestations in patients with COVID-19. Available data do not suggest specific focus on the mucous membranes and the mouth.

Biadsee 2020

	Yes	No	Unclear	Not applicable
1. Was the sample frame appropriate to address the target population?	X			
2. Were study participants sampled in an appropriate way?	X			
3. Was the sample size adequate?	X			
4. Were the study subjects and the setting described in detail?	X			
5. Was the data analysis conducted with sufficient coverage of the identified sample?	X			
6. Were valid methods used for the identification of the condition?		X		
7. Was the condition measured in a standard, reliable way for all participants?		X		
8. Was there appropriate statistical analysis?				X
9. Was the response rate adequate, and if not, was the low response rate managed appropriately?	X			

Overall appraisal: Include X Exclude Seek further info

Comments (Including reason for exclusion):

Fantozzi 2020

	Yes	No	Unclear	Not applicable
1. Was the sample frame appropriate to address the target population?	X			
2. Were study participants sampled in an appropriate way?	X			
3. Was the sample size adequate?	X			
4. Were the study subjects and the setting described in detail?	X			
5. Was the data analysis conducted with sufficient coverage of the identified sample?	X			
6. Were valid methods used for the identification of the condition?		X		
7. Was the condition measured in a standard, reliable way for all participants?		X		

8. Was there appropriate statistical analysis?				X
9. Was the response rate adequate, and if not, was the low response rate managed appropriately?	X			

Overall appraisal: Include Exclude Seek further info

Comments (Including reason for exclusion):

Fidan 2021

	Yes	No	Unclear	Not applicable
1. Was the sample frame appropriate to address the target population?	X			
2. Were study participants sampled in an appropriate way?	X			
3. Was the sample size adequate?		X		
4. Were the study subjects and the setting described in detail?	X			
5. Was the data analysis conducted with sufficient coverage of the identified sample?	X			
6. Were valid methods used for the identification of the condition?	X			
7. Was the condition measured in a standard, reliable way for all participants?	X			
8. Was there appropriate statistical analysis?				X
9. Was the response rate adequate, and if not, was the low response rate managed appropriately?	X			

Overall appraisal: Include X Exclude Seek further info

Comments (Including reason for exclusion):

Gherlone 2021

	Yes	No	Unclear	Not applicable
1. Was the sample frame appropriate to address the target population?	X			
2. Were study participants sampled in an appropriate way?	X			
3. Was the sample size adequate?				
4. Were the study subjects and the setting described in detail?	X			
5. Was the data analysis conducted with sufficient coverage of the identified sample?	X			
6. Were valid methods used for the identification of the condition?	X			
7. Was the condition measured in a standard, reliable way for all participants?	X			

8. Was there appropriate statistical analysis?				X
9. Was the response rate adequate, and if not, was the low response rate managed appropriately?	X			

Overall appraisal: Include X Exclude Seek further info

Comments (Including reason for exclusion):

Khabadze 2020

	Yes	No	Unclear	Not applicable
1. Was the sample frame appropriate to address the target population?	X			
2. Were study participants sampled in an appropriate way?	X			
3. Was the sample size adequate?		X		
4. Were the study subjects and the setting described in detail?	X			
5. Was the data analysis conducted with sufficient coverage of the identified sample?	X			
6. Were valid methods used for the identification of the condition?			X	
7. Was the condition measured in a standard, reliable way for all participants?			X	
8. Was there appropriate statistical analysis?				X
9. Was the response rate adequate, and if not, was the low response rate managed appropriately?	X			

Overall appraisal: Include Exclude X Seek further info

Comments (Including reason for exclusion): Available data mention oral conditions that most likely are concurrent with COVID-19 and not related to this particular infection (plaque on the tongue and gingival pigmentation). No data are available on specific oral lesions.

Nuno-Gonzalez 2021

	Yes	No	Unclear	Not applicable
1. Was the sample frame appropriate to address the target population?	X			
2. Were study participants sampled in an appropriate way?	X			
3. Was the sample size adequate?	X			
4. Were the study subjects and the setting described in detail?	X			
5. Was the data analysis conducted with sufficient coverage of the identified sample?	X			
6. Were valid methods used for the identification of the condition?		X		

7. Was the condition measured in a standard, reliable way for all participants?		X		
8. Was there appropriate statistical analysis?				X
9. Was the response rate adequate, and if not, was the low response rate managed appropriately?	X			

Overall appraisal: Include X Exclude Seek further info

Comments (Including reason for exclusion):

Rekhtman 2020

	Yes	No	Unclear	Not applicable
1. Was the sample frame appropriate to address the target population?	X			
2. Were study participants sampled in an appropriate way?	X			
3. Was the sample size adequate?				
4. Were the study subjects and the setting described in detail?	X			
5. Was the data analysis conducted with sufficient coverage of the identified sample?	X			
6. Were valid methods used for the identification of the condition?		X		
7. Was the condition measured in a standard, reliable way for all participants?		X		
8. Was there appropriate statistical analysis?				X
9. Was the response rate adequate, and if not, was the low response rate managed appropriately?	X			

Overall appraisal: Include Exclude X Seek further info

Comments (Including reason for exclusion): The study aimed to study eruptions on all body parts in patients with COVID-19. Available data do not suggest focus on the mouth.

Riad 2020

	Yes	No	Unclear	Not applicable
1. Was the sample frame appropriate to address the target population?	X			
2. Were study participants sampled in an appropriate way?	X			
3. Was the sample size adequate?	X			
4. Were the study subjects and the setting described in detail?	X			
5. Was the data analysis conducted with sufficient coverage of the identified sample?	X			
6. Were valid methods used for the identification of the condition?		X		

7. Was the condition measured in a standard, reliable way for all participants?		X		
8. Was there appropriate statistical analysis?				X
9. Was the response rate adequate, and if not, was the low response rate managed appropriately?	X			

Overall appraisal: Include X Exclude Seek further info

Comments (Including reason for exclusion): The study aimed to study eruptions on all body parts in patients with COVID-19. Available data do not suggest focus on the mouth.
